# Factors influencing degradation kinetics of mRNAs and half-lives of microRNAs, circRNAs, lncRNAs in blood in vitro using quantitative PCR

**DOI:** 10.1038/s41598-022-11339-w

**Published:** 2022-05-04

**Authors:** Chong Wang, Hui Liu

**Affiliations:** College of Medical Laboratory, Dalian Medical University, Dalian, 116044 China

**Keywords:** Biochemistry, Genetics, Molecular biology

## Abstract

RNAs are rapidly degraded in samples and during collection, processing and testing. In this study, we used the same method to explore the half-lives of different RNAs and the influencing factors, and compared the degradation kinetics and characteristics of different RNAs in whole blood and experimental samples. Fresh anticoagulant blood samples were incubated at room temperature for different durations, RNAs were extracted, and genes, including internal references, were amplified by real-time quantitative PCR. A linear half-life model was established according to cycle threshold (Ct) values. The effects of experimental operations on RNA degradation before and after RNA extraction were explored. Quantitative analysis of mRNA degradation in samples and during experimental processes were explored using an orthogonal experimental design. The storage duration of blood samples at room temperature had the greatest influence on RNA degradation. The half-lives of messenger RNAs (mRNAs) was 16.4 h. The half-lives of circular RNAs (circRNAs), long non-coding RNAs (lncRNAs) and microRNAs (miRNAs) were 24.56 ± 5.2 h, 17.46 ± 3.0 h and 16.42 ± 4.2 h, respectively. RNA degradation occurred mainly in blood samples. The half-life of mRNAs was the shortest among the four kinds of RNAs. Quantitative experiments related to mRNAs should be completed within 2 h. The half-lives of circRNAs and lncRNAs were longer than those of the former two.

## Introduction

Blood samples are rich in biomolecules such as proteins, DNA and RNA, which can be used for disease diagnosis, and determining disease stage and prognosis^1^. Compared with tissue samples, blood samples have advantages including being more readily accessible, suitable for continuous sampling, and amenable to numerous different tests. However, after blood samples are collected, they need to be processed, packed and stored in containers suitable for long-term cryopreservation. Otherwise, RNA in blood is prone to degradation under normal temperature transportation and daily storage conditions, which can affect the subsequent detection, analysis and development of molecular diagnostic biomarkers^2^.

In clinical diagnostic laboratories, 60–70% of errors occur in the pre-processing process of specimen analysis, most of which are caused by specimen collection, specimen processing and specimen storage^3^. As a basis for biomarker research and development, the acquisition of high quality blood samples is critical. The dipotassium ethylene diamine tetraacetic acid (EDTA-2K) anticoagulant vessels are widely used in basic research and clinical laboratories to collect, transport and preserve blood samples, which is not conducive to protecting specific molecular biomarkers such as RNA molecules in blood. In the process of sample preservation, if a sample is immediately frozen at − 20 °C or − 80 °C without any protective measures, subcellular structures are severely damaged, and the structure and activity of RNA can be altered, often irreversibly^4^. The quality of RNA in stored samples is directly affected by the method of sample storage^5^. In the case of improper storage, RNA in blood is rapidly degraded by RNase enzymes present in blood or the external environment. Previous research has shown that although EDTA-K2 can effectively prevent hemagglutination, it cannot maintain the stability of RNA in blood cells. Following cryopreservation, RNA is degraded by about 80% in a short time^6^. Therefore, the present work mainly compared the stability of messenger RNAs (mRNAs) and some non-coding RNAs (ncRNAs) in blood, explored the influence of relevant experimental operations on RNA degradation before and after RNA extraction, and quantitatively analysed on mRNA degradation in specimens and during experimental processes.

The miRNAs are short (~ 22 nucleotides), non-coding, RNA molecules that control diverse biological processes, including cell fate determination, cell proliferation, cell differentiation and cell death^7–9^. The miRNAs regulate gene expression post-transcriptionally by interacting with and down-regulating target mRNA molecules. They are relatively stable and widely expressed in cells, tissues and organs throughout the body. In serum, plasma, saliva, urine, emulsions and other body fluids, miRNAs are highly stable extracellularly^10–12^. The miRNAs are associated with a variety of diseases and have been detected under a variety of pathological conditions^13^. CircRNAs are a common type of non-coding RNA produced by reverse splicing. Reverse splicing is an unconventional splicing event characterized by non-coding RNA molecules with circular structure formed by covalent bonds without a 5′-capped structure or a 3′-terminated polyadenylated tail. Since circRNAs are insensitive to nuclease, they are more stable than linear RNAs, but the mechanism by which cells eventually degrade circRNAs is still largely unknown compared to the mechanism of circRNA biogenesis^14,15^. LncRNAs are non-coding RNAs with a length of more than 200 nucleotides. Studies have confirmed the existence of hundreds of lncRNAs in serum/plasma. These long-chain molecules are stable and abundant, easy to be quantitatively detected, and have significant disease specificity^13^.

Although great progress has been made on studying ncRNAs biosynthesis and functions, there are few reports on their degradation^16^. Due to the presence of nucleases in the blood, most researchers doubt that extracellular RNA can remain stable for long periods of time^17^. For example, the miRNAs are generally considered to have a long half-life (T_1/2_), and can be stably present in serum and not easily degraded. Like other RNAs, miRNAs have a half-life, but exact data is rare in the literature^18,19^. The regulation of miRNAs is critical to the definition of cell identity and behaviour in normal physiology and disease. However, the dynamics of miRNA degradation and the mechanisms involved remain largely obscure, particularly in higher organisms^20^.

The determination of RNA half-life (T_1/2_) is important for understanding the regulatory mechanisms of gene expression and environmental changes that alter gene transcription levels^21^. Each method for measuring RNA levels has its advantages and disadvantages. The half-life of mRNA in tissues is generally determined by in situ hybridisation, which is time-consuming and labour-intensive^9^. Plasma ncRNAs can be quantitatively detected by real-time fluorescence quantitative PCR and microarray. If higher sensitivity is required, real-time quantitative PCR is a good choice^9,22^. The copy number of molecules in samples can be quickly and accurately determined by real-time quantitative PCR. The relative initial concentration is calculated based on the cycle threshold (Ct) value, and the half-life is calculated by establishing the regression linear equation over time. Due to the sensitivity of the method, the half-life of an RNA can be established even when it is expressed at low levels.

Therefore, a fast and reliable RNA half-life measurement method was established in the present study, based on real-time fluorescence quantitative PCR and supplemented by microspectrophotometry, to compare the half-life length of mRNAs and ncRNAs. Human-specific glyceraldehyde-3-phosphate dehydrogenase (GAPDH) and β-actin genes were used as internal reference genes for mRNA stability since their expression levels in cells or copy number in the genome is constant, and is less affected by environmental factors^23^. The rest of the genes were randomly selected according to literatures: the miR-221, miR-16-1, miR-126, miR-145 and miR-28-3p genes were used as observed miRNA^24–28^; the hsa_circRNA_002532, hsa_circ_0000190, hsa_circ_0001785, hsa_circ_0000520 and circARIDIB genes were used as observed circRNA^29–31^ and lncRNA GASL, lncRNA PCGEM1, STEAP3-AS1, NR-038263 and LncRNA SNHG5 were used as observed lncRNA^32–34^. Changes in RNA content in blood samples after different time periods were determined by fluorescence quantitative PCR, and the half-lives of the genes were established and assessed. Fresh, whole anticoagulant blood was collected and stored at room temperature, and RNA was extracted and measured every 12 h. Real-time quantitative PCR analysis of the genes was used to evaluate the concentrations of mRNAs and ncRNAs in each sample. We analysed various factors involved, from RNA extraction to gene amplification. We employed the orthogonal experimental method with three factors and three levels. The three factors were the storage time of fresh whole blood at room temperature, the storage time of RNA at room temperature, and the storage time of cDNA after reverse transcription at − 20 °C. The β-actin gene was amplified by real-time quantitative PCR and its Ct value was determined. The results were analysed by multivariate analysis of variance (ANOVA) and regression analysis^35^.

The present work focused on exploring the degradation of RNA at room temperature, using the same method to study the half-life of different RNAs and the influencing factors, and compare the degradation kinetics and characteristics of different RNAs in whole blood. The overall aim was to establish the optimal experimental and storage conditions, and thereby ensure the accuracy of experimental results.

## Materials and methods

### Treatment of experimental specimens

Fresh anticoagulant whole blood was used as experimental samples. Next, genomic RNA was extracted using a high efficiency blood RNA extraction kit (Beijing TIANGEN Biotechnology, batch number DP190813) according to the instructions supplied with the kit. After extraction, RNA concentration and purity were calculated using a microspectrophotometer (Vastech Inc.Wilmington, USA) according to the absorbance at 260 and 280 nm (A260 and A280). Reverse transcription was carried out in a 20 µL system with primers and reverse transcriptase (The system contains primers, RNA templates, 2 × ES Reaction Mix, RT Enzyme Mix and gDNA Remover). A 1 µL sample of the reverse transcription product was used for real-time quantitative PCR, and each gene was amplified by PCR. Primer sequences are shown in Table 1. Real-time quantitative PCR reaction system is shown in Table 2.

**Table 1:** Primer sequences of genes.

Primer names	Length (mer)	Sequence (5′–3′)
GAPDH-F	19	CCTCAAGATCATCAGCAAT
GAPDH-R	20	CCATCCACAGTCTTCTGGGT
β-Actin-F	18	CCAGGTCATCACCATCGG
β-Actin-R	18	TGTCCACGTCGCACTTCA
LncRNA GASL1-F	20	CTGAGGCCAAAGTTTCCAAC
LncRNA GASL1-R	21	CAGCCTGACTTTCCCTCTTCT
lncRNA PCGEM1-F	20	ACCTTTTTGCCCTATGCCGT
lncRNA PCGEM1-R	20	ACGTTGAGTCCCAGTGCATC
STEAP3-AS1-F	20	TGCTGGGAAAGGGAACTCTG
STEAP3-AS1-R	21	TCCTGGTCATCAAACACCCAG
NR-038263-F	22	TATTGGCAGGCTACACCTAAGA
NR-038263-R	20	TGCGGATTTAGAGTGAGGTG
LncRNA SNHG5-F	18	TACTGGCTGCGCACTTCG
LncRNA SNHG5-R	19	CAGTAAAAGGGGAACACCA
circRNA002532-F	21	TGGGAGTTTTCTGCTGATGAT
circRNA002532-R	23	GGGTTTCTTTCTCATCTCTCTCA
hsa_circ_0000190-F	20	TTGCTCCTTGGGCGCTATAC
hsa_circ_0000190-R	21	AGAGTCCAGCGGCAAAACTA
hsa_circ_0001785-F	20	CAGTTTTTGATTGCCCCTCC
hsa_circ_0001785-R	20	GTGTCGTGGGTCTAGTAACC
hsa_circ_0000520-F	22	GGAAGGTCTGAGACTAGGGCCA
hsa_circ_0000520-R	21	AAGGGACATGGGAGTGGAGTG
circARIDIB-F	19	CTCGATCTGGCCCAATCTC
circARIDIB-R	18	CCAAAGGCTGCATCCTCC
hsa-miR-221-RT	50	GTCGTATCCAGTGCAGGGTCCGAGGTATTCGCACTGGATACGACgaaacc
hsa-miR-221-F	22	GGAGCTACATTGTCTGCTGGG
hsa-miR-16-1-RT	50	GTCGTATCCAGTGCAGGGTCCGAGGTATTCGCACTGGATACGACtcagca
hsa-miR-16-1-F	21	GGGCCCAGTATTAACTGTGCTG
hsa-miR-126-RT	50	GTCGTATCCAGTGCAGGGTCCGAGGTATTCGCACTGGATACGACcgcatt
hsa-miR-126-F	22	GCGCTCGTACCGTGAGTAATAA
hsa-miR-28-3p-RT	50	GTCGTATCCAGTGCAGGGTCCGAGGTATTCGCACTGGATACGACtccagg
hsa-miR-28-3p-F	21	GCGCACTAGATTGTGAGCTCC
hsa-miR-145-RT	50	GTCGTATCCAGTGCAGGGTCCGAGGTATTCGCACTGGATACGACagaaca
hsa-miR-145-F	21	CCCGGATTCCTGGAAATACTG
Universal R	19	CCAGTGCAGGGTCCGAGGT
snRNA U6-F	17	CTCGCTTCGGCAGCACA
snRNA U6-R	20	AACGCTTCACGAATTTGCGT

**Table 2:** Real-time fluorescence quantitative PCR reaction system.

Component	Volume (μl)
RNase-free ddH2O	8.2
Template	1
Forward primer	0.4
Reverse primer	0.4
2 × TransStart Top Green qPCR SuperMix	10
Total volume	20

(1) Predenaturation at 94 °C for 30 s; (2) Denaturation at 94 °C for 5 s, annealing at 60 °C for 15 s, extension at 72 °C for 10 s, circulating 40 times.

### Quantitative performance evaluation of detection system

#### Detection using a high efficiency blood total RNA extraction kit

Whole blood was diluted to a series of concentrations, RNA was extracted using a high efficiency total blood RNA extraction kit, and a linear relationship between RNA concentration and dilution ratio was observed. The experiment was repeated three times.

#### Detection using a microspectrophotometer

Extracted RNA was diluted to a series of concentrations and concentration was measured with a microspectrophotometer, revealing a linear relationship between RNA concentration and dilution ratio. The experiment was repeated three times.

#### Real-time fluorescence quantitative PCR

Extracted RNA was reverse-transcribed into cDNA and diluted to a series of concentrations. The GAPDH gene was amplified by real-time quantitative PCR, and a linear relationship between its Ct value and dilution ratio was observed. The experiment was repeated three times.

### The influence of three factors on RNA degradation rate determined by an orthogonal method

Three factors and three levels of orthogonal experimental methods were employed. The three factors were the storage time of fresh whole blood at room temperature, the storage time of RNA at room temperature, and the storage time of cDNA after reverse transcription at − 20 °C. The Ct values of internal reference genes were determined by real-time quantitative PCR and the results were analysed by multivariate ANOVA and regression analysis. The optimal combination was obtained according to the results of orthogonal experiments. Whole blood was diluted 1:2, 1:4, 1:8, 1:16 and 1:32 with normal saline. RNA extraction and other related experiments were carried out under optimal combination conditions. GAPDH, β-actin and U6 genes were amplified by real-time quantitative PCR.

### Comparison of the half-lives of mRNAs and ncRNAs

When whole blood samples were incubated at room temperature, the RNA concentration gradually decreased with time. The half-life was calculated using a microspectrophotometer and real-time fluorescence quantitative PCR. Firstly, according to the equation N_t_ = 2^(Ct0−Ctt)^, the relative template concentration (N_t_) was calculated from the Ct values (t represents the t_th_ 12-h period of incubation after the specimen is placed; Ct_0_ represents the Ct value at 0 h of specimen placement; Ctt represents the Ct value of the specimen after it has been placed for a period of time). K derived from the linear regression equation between the natural logarithm of the concentrations (N_t_) and the incubation time, is the slope of the equation. The half-life(T_1/2_) was obtained using the equation^36,37^:

$$\mathrm{T}1/2=\mathrm{ln}2/|\mathrm{k}|$$

For example, the degradation of RNA in blood samples: the samples are incubated for 0, 12, 24, 36, 48, 60 h in order (Times), and the corresponding Ct values are 22.04, 22.34, 23.41, 24.15, 24.40, 25.78, respectively, and the corresponding concentration (N_t_) are 1.00, 0.81, 0.39, 0.23, 0.19, 0.07, respectively, and then the natural logarithm of the concentration (lnN_t_) is transformed as 0, − 0.21, − 0.95, − 1.46, − 1.64, − 2.59 respectively. The linear regression is analysed using lnN_t_ against incubation time, and the regression equation (y = − 0.0423x + 0.1262) is obtained, the slope (k) = 0.0423. Therefore according to the formula T_1__/2_ = ln2/|k|, the half-life (T_1/2_) is 16.4 h.

### Statistical analysis

All data were analysed using SPSS17.0 statistical software^38^. Multi-factor ANOVA was performed on the orthogonal test results. The storage time of fresh whole blood at room temperature, the storage time of RNA at room temperature, and the time of reverse transcription after cDNA storage at − 20 °C were considered as independent variables, and the concentration was taken as a dependent variable to investigate the effects of the three factors on RNA degradation at different levels. A *p*-value < 0.05 indicated statistically significant differences.

## Results

**Figure 1:**
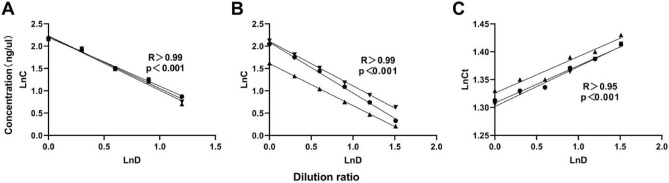
Linear detection using a high-efficiency blood RNA extraction kit (**A**). Linear detection by microspectrophotometry (**B**). Linear detection by real-time quantitative PCR amplification (**C**).

In order to evaluate the quantitative performance of the detection system, linear observation was carried out. The results showed revealed a good linear relationship between the natural logarithm of the concentration value, the Ct value, and the natural logarithm of the dilution factor, with correlation coefficient R of 0.9972, 0.9931, 0.9925, 0.9987, 0.9997, 0.9993, 0.9892, 0.9759 and 0.9914, respectively, all greater than 0.95 (Fig. 1). After statistical calculation, *p*-values of the three groups were all less than 0.001, showing statistical differences. Therefore, the experimental results obtained from subsequent experiments based on the above experimental instruments were reliable.

### Orthogonal tests

Nine experiments were performed according to the L_9_(3^4^) orthogonal experimental table of three factors and three levels, with each factor at three levels. The β-actin gene was amplified by real-time quantitative PCR to measure the Ct value. The results are shown in Table 3.

**Table 3:** L_9_(3^4^) orthogonal experiment.

Test	A	B	C	β-Actin (Ct)	Concentration
1	0 h	0 h	0 h	21.99	1.000
2	0 h	12 h	12 h	22.62	0.646
3	0 h	24 h	24 h	22.73	0.599
4	12 h	0 h	12 h	23.71	0.304
5	12 h	12 h	24 h	23.73	0.299
6	12 h	24 h	0 h	23.43	0.369
7	24 h	0 h	24 h	24.34	0.196
8	24 h	12 h	0 h	24.15	0.224
9	24 h	24 h	12 h	24.39	0.189

A represents the storage time of fresh whole blood at room temperature; B represents the storage time of RNA at room temperature; C represents the storage time of cDNA at − 20 °C.

Direct comparison of the nine experimental results in Table 3 shows that the Ct value of Experiment 1 was the smallest. With increasing time, the Ct value of genes gradually increased (i.e., the more factors involved, the greater the impact on RNA degradation, and the faster RNA degradation). Multivariate ANOVA and linear regression analysis were conducted for the above orthogonal experiments, and the analysis results are shown in Tables 4 and 5.

**Table 4:** Analysis of between-sample effects.

Source	Type III sum of squares	F	p
Corrected model	1.446^a^	39.588	< 0.001
Intercept	4.724	775.970	< 0.001
A	1.286	105.598	< 0.001
B	0.048	3.905	0.037
C	0.113	9.261	< 0.001
a.R Squared = 0.922			

Dependent variable = concentration. A represents the storage time of fresh whole blood at room temperature; B represents the storage time of RNA at room temperature; C represents the storage time of cDNA at − 20 °C.

**Table 5:** The coefficient of linear regression.

Model	Unstandardized coefficients	Standardized coefficients	t	p
	B	Beta		
(Constant)	1.251		6.601	0.001
A	− 0.273	− 0.868	− 5.121	0.004
B	− 0.057	− 0.182	− 1.074	0.332
C	− 0.083	− 0.264	− 1.559	0.180

A represents the storage time of fresh whole blood at room temperature; B represents the storage time of RNA at room temperature; C represents the storage time of cDNA at − 20 °C.

In the table comparing inter-subject effects, we compared the *p*-values of fresh whole blood storage time at room temperature, RNA storage time at room temperature, and cDNA storage time at − 20 °C, and the *p*-values of the three influencing factors were all less than 0.05, and less than 0.01 for two of the influencing factors. Thus, these three factors had significant effects on RNA degradation, and the differences were statistically significant. However, there were some differences in the degree of influence. According to the regression analysis coefficient results, the storage time of RNA at room temperature had the least influence on RNA degradation, while the storage time of fresh whole blood at room temperature had the largest influence on RNA degradation. The effects of the three factors on RNA degradation were ordered whole blood storage time at room temperature > cDNA storage time at − 20 °C > RNA storage time at room temperature.

According to the results of orthogonal experiments, the optimal combination of factors in this experiment was A1B1C1 (i.e., when the storage time of fresh whole blood at room temperature, the storage time of RNA at room temperature, and the storage time of reverse transcription cDNA at − 20 °C were all 0 h, the Ct value of genes was lowest). Therefore, in the process of RNA extraction and reverse transcription from whole blood, the time should be shortened as soon as possible, otherwise the subsequent results will be affected to a certain extent. Furthermore, blood was diluted to a series of concentrations for RNA extraction, followed by the amplification of GAPDH, β-actin, and U6 genes, and Ct values were recorded, all under optimal conditions. Taking the natural logarithm of the relative template concentration as a linear relationship with the dilution ratio, the correlation coefficient R values were 0.95, 0.99 and 0.93, respectively, all greater than 0.9, showing a good linear relationship.

### Half-life calculation

For half-life calculation based on microspectrophotometry, fresh anticoagulant whole blood was placed at room temperature, RNA was extracted every 12 h, and the concentration was measured by microspectrophotometry. The results are shown in Fig. S1A. Linear regression analysis of the natural logarithm of concentration and time yielded the concentration equation y = − 0.0481x + 4.172, R = 0.972 (Fig. S1B), and the calculated half-life was 14.4 h.

**Figure 2:**
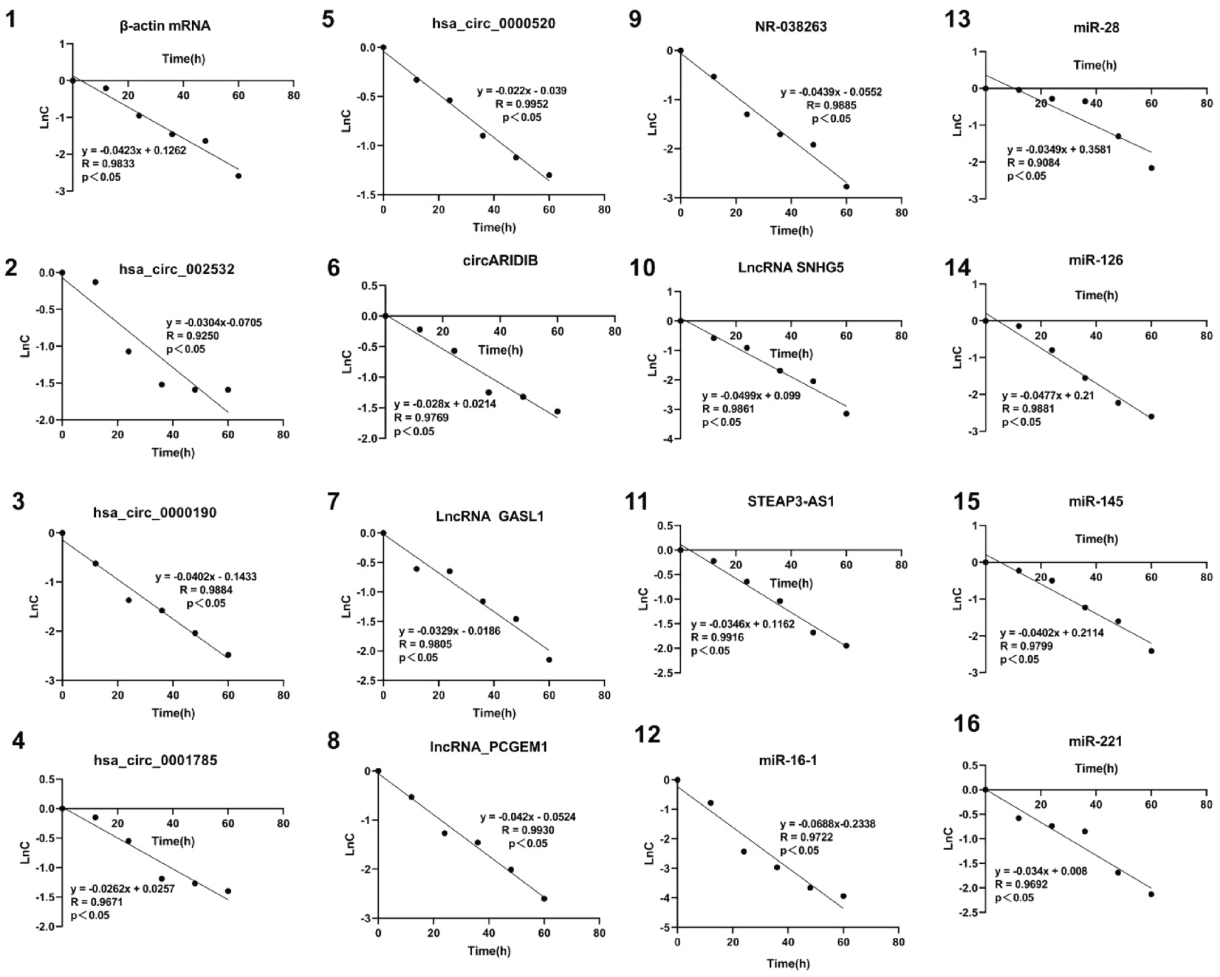
Dynamic degradation curves for mRNA, circRNAs, lncRNAs and miRNAs in blood after natural logarithm transformation.

For half-life calculation based on the real-time fluorescence quantitative PCR method, the degradation data at room temperature are shown in Tables 6, 7 and 8. The RNA concentration in whole blood decreased with increasing incubation duration. The half-life was calculated by performing linear regression analysis on the natural logarithm of the initial concentration and the incubation duration. The regression analysis diagram is shown in Fig. 2. The correlation coefficients (R) of all linear fitting equations are greater than 0.9. The R and the equations were shown in Table 9. And p-values of each group were all less than 0.05, showing statistical differences. The half-lives of mRNA and miRNA in whole blood were calculated by the formula to be 16.4 h and 16.42 ± 4.2 h, respectively, indicating that the half-lives of mRNA were similar to those of miRNA. The half-lives of circRNA and lncRNA in whole blood were 24.56 ± 5.2 h and 17.46 ± 3.0 h, respectively. When the confidence level is 95%, the confidence interval of miRNA, circRNA and lncRNA half-life is (12.739, 20.101), (20.002, 29.118) and (14.830, 20.090) respectively, as shown in Tables 6, 7 and 8.

**Table 6:** Changes in circRNAs concentration in whole blood at room temperature.

Gene	Time(h)								
	0 h	12 h	24 h	36 h	48 h	60 h	T_1/2_	Products length	$\overline{x }$ ± s
**Circ002532**									24.56 ± 5.2 h
Ct	26.43	26.62	27.98	28.63	28.72	28.73	22.80 h	119 bp	
C	1.00	0.88	0.34	0.22	0.20	0.20			
**hsa_circ_0000190**									
Ct	28.30	29.19	30.28	30.58	31.24	31.88	17.24 h	122 bp	
C	1.00	0.54	0.25	0.21	0.13	0.08			
**hsa_circ_0001785**									
Ct	29.11	29.32	29.91	30.83	30.94	31.13	26.50 h	135 bp	
C	1.00	0.86	0.57	0.30	0.28	0.25			
**hsa_circ_0000520**									
Ct	27.35	27.82	28.13	28.65	28.97	29.22	31.51 h	148 bp	
C	1.00	0.72	0.58	0.41	0.33	0.27			
**circARIDIB**									
Ct	28.22	28.54	29.04	30.02	30.12	30.47	24.76 h	71 bp	
C	1.00	0.80	0.57	0.29	0.27	0.21			

The 95% confidence interval for T_1/2_ was calculated to be (20.002, 29.118).

**Table 7:** Changes in lncRNAs concentration in whole blood at room temperature.

Gene	Time(h)								
	0 h	12 h	24 h	36 h	48 h	60 h	T_1/2_	Products length	$\overline{x }$ ± s
**LncRNA GASL**									17.46 ± 3.0 h
Ct	32.53	33.41	33.47	34.21	34.64	35.63	21.10 h	210 bp	
C	1.00	0.54	0.52	0.31	0.23	0.12			
**lncRNA PCGEM1**									
Ct	30.53	31.30	32.36	32.64	33.43	34.28	16.50 h	156 bp	
C	1.00	0.59	0.28	0.23	0.13	0.07			
**STEAP3-AS1**									
Ct	25.40	25.72	26.32	26.90	27.83	28.21	20.03 h	149 bp	
C	1.00	0.80	0.53	0.35	0.19	0.14			
**NR-038263**									
Ct	27.47	28.23	29.34	29.93	30.24	31.46	15.79 h	225 bp	
C	1.00	0.59	0.27	0.18	0.15	0.06			
**LncRNASNHG5**									
Ct	26.38	27.21	27.69	28.82	29.34	30.92	13.89 h	101 bp	
C	1.00	0.56	0.40	0.18	0.13	0.04			

The 95% confidence interval for T_1/2_ was calculated to be (14.830, 20.090).

**Table 8:** Changes in miRNAs concentration in whole blood at room temperature.

Gene	Time(h)								
	0 h	12 h	24 h	36 h	48 h	60 h	T_1/2_	Products length	$\overline{x }$ ± s
**miR-221**									16.42 ± 4.2 h
Ct	18.45	19.28	19.52	19.68	20.89	21.52	20.39 h	65 bp	
C	1.00	0.56	0.48	0.43	0.18	0.12			
**miR-16-1**									
Ct	18.33	19.45	21.83	22.62	23.61	24.01	10.10 h	61 bp	
C	1.00	0.46	0.09	0.05	0.03	0.02			
**miR-126**									
Ct	23.32	23.52	24.47	25.56	26.53	27.07	14.53 h	63 bp	
C	1.00	0.87	0.45	0.21	0.11	0.07			
**miR-145**									
Ct	20.94	21.27	21.66	22.72	23.25	24.41	17.24 h	65 bp	
C	1.00	0.80	0.61	0.29	0.20	0.09			
**miR-28-3p**									
Ct	24.69	24.75	25.09	25.20	26.56	27.81	19.86 h	60 bp	
C	1.00	0.96	0.76	0.70	0.27	0.12			

The 95% confidence interval for T_1/2_ was calculated to be (12.739, 20.101).

**Table 9:** Linear regression analysis of different genes.

Gene	Linear regression equations	Correlation coefficients R	p value
β-Actin mRNA	y = − 0.0423x + 0.1262	0.9833	< 0.001
Circ002532	y = − 0.0304x − 0.0705	0.9250	0.008
hsa_circ_0000190	y = − 0.0402x − 0.1433	0.9884	< 0.001
hsa_circ_0001785	y = − 0.0262x + 0.0257	0.9671	0.002
hsa_circ_0000520	y = − 0.0220x − 0.039	0.9952	< 0.001
circARIDIB	y = − 0.0280x + 0.0214	0.9769	0.001
LncRNA GASL	y = − 0.0329x − 0.0186	0.9805	< 0.001
lncRNAPCGEM1	y = − 0.0420x − 0.0524	0.9930	< 0.001
STEAP3-AS1	y = − 0.0346x + 0.1162	0.9916	< 0.001
NR-038263	y = − 0.0439x − 0.0552	0.9885	< 0.001
LncRNA SNHG5	y = − 0.0499x + 0.099	0.9861	< 0.001
miR-221	y = − 0.0340x + 0.008	0.9692	0.001
miR-16-1	y = − 0.0688x − 0.2338	0.9722	0.001
miR-126	y = − 0.0477x + 0.21	0.9881	< 0.001
miR-145	y = − 0.0402x + 0.2114	0.9799	0.001
miR-28-3p	y = − 0.0349x + 0.3581	0.9084	0.012

## Discussion

In this study, a fast and reliable RNA half-life measurement method was established, based on quantitative real-time fluorescence PCR and supplemented by microspectrophotometry. Quantitative real-time fluorescence PCR can quickly and accurately determine the copy number of molecules in samples. qPCR was employed to detect changes in RNA content after incubating samples for different time periods, and the half-lives of mRNA and ncRNAs were compared^39^. Because incomplete or degraded RNA may interfere with the accuracy of RNA half-life detection, the length of the amplified fragment is important to determine the RNA half-life^40^. Regarding genes, we selected β-actin, GAPDH and other housekeeper genes as internal reference sequences. Since their expression levels in cells or copy number in the genome is constant, and is less affected by environmental factors, their quantitative results reflect the number of cells or genomes contained in the sample^41^.

In order to avoid errors caused by manual operation and differences in reagents and instruments, we conducted quantitative performance evaluation of the methods involved in the experimental process before the experiment. And the correlation coefficient R values were greater than 0.95, with good reliability (Fig. 1), and these good linear relationships laid a foundation for subsequent experiments.

In the analysis of key influencing factors, we employed an orthogonal experiment to investigate multiple factors accurately. The approach uses a set of normalised orthogonal tables to conduct tests, and the experimental results are subjected to statistical analysis to draw scientific conclusions. This design method is used for studying multi-factors and multi-levels rapidly and conveniently^42^. The orthogonal experiment results showed that the greater the number of factors involved in the experiment, the greater the impact on RNA degradation, and the faster RNA degradation. Multivariate ANOVA in the orthogonal experiment showed that the *p*-values of the three influencing factors were all less than 0.05, and *p*-values for two of the influencing factors were less than 0.01 (Table 4), indicating that the three factors had a significant impact on RNA degradation, and the differences were statistically significant. By comparing the standardisation coefficient, it could be seen that the storage duration of RNA at room temperature had the least effect on RNA degradation, while the storage duration of fresh whole blood at room temperature had the largest effect on RNA degradation. Therefore, we studied the storage duration as a factor, and investigated the half-life of mRNA, miRNA, lncRNA and circRNA in whole blood at room temperature.

When processing actual samples, especially during mass inspection, experimentalists often ignore the effects of intermediate processes from specimen collection to specimen treatment on RNA concentration^3^. Therefore, we chose room temperature as the most likely incubation temperature for blood samples after collection. In view of the fact that the target gene fragment could not be amplified after whole blood samples were incubated for a week in preliminary experiments, we tested observation times of 0 h, 12 h, 24 h, 36 h, 48 h, and 60 h. The half-life was calculated by two methods (microspectrophotometry and qPCR). First, RNA concentration was measured by microspectrophotometry, we found that RNA degradation was fastest in the first 24 h (the slope of the dotted line was the greatest), and the slope of the dotted line became more gentle after 24 h. This shows that RNA is particularly prone to degradation which is consistent with literature reports^43,44^. The linear equation was calculated and the half-life of whole blood RNA at room temperature was 14.4 h. Second, using real-time quantitative PCR, the relative concentration was calculated according to the formula after taking the median Ct value, linear regression analysis was performed on the natural logarithm of the concentration vs. time, and the correlation coefficients were all greater than 0.9 (Table 9). Therefore, we can say that at room temperature, the degradation of RNA in whole blood after a defined period of time conforms to the first-order kinetic law. The experimental results showed that the half-life of mRNA was 16.4 h, and the half-life of miRNA was 16.42 ± 4.2 h. Thus, the half-life of mRNA was shorter than that of miRNA, but only slightly. The half-lives of circRNA and lncRNA were longer than those of the other two. Therefore, circRNAs are relatively stable, which is consistent with literature reports^45,46^. However, we found that there seems to be no relationship between the length of the amplified fragments and stability because even though the amplified fragments are similar in length, their half-lives are still very different (Tables 6, 7, 8).

In this experiment, the half-life results obtained by real-time fluorescence quantitative PCR and microspectrophotometry were essentially similar, and mRNAs and ncRNAs were measured simultaneously on the same platform. Therefore, we concluded that the storage method for samples directly affected the quality of RNA in the stored samples. RNA in blood is easily degraded during improper storage, and RNA is rapidly degraded by RNases present in blood or in the external environment. Storage at room temperature has a greater impact on RNA degradation^47^.

The mRNAs and ncRNAs studied in the present work were from blood samples. Most previous studies have focused on tissue samples, but blood samples have advantages including being clearer, non-invasive, convenient for sampling, and amenable to continuous detection^1^. With the development of biotechnology, detection methods will become more and more sensitive, hence it is particularly important to reduce variation during pre-treatment of specimens^48^. Due to the poor stability and short half-life of RNA, it is of great significance to select appropriate and professional methods to preserve blood samples to ensure the accuracy and reliability of test results. Especially in clinical trials, the acquisition of high-quality experimental samples is very important, hence more attention should be paid to the impacts of specimen analysis and processing^49^.

## Supplementary Information

Supplementary Information.

## Data Availability

The datasets used and/or analysed during the current study are available from the corresponding author on reasonable request.

## Abbreviations

Ct: Cycle threshold
miRNAs: MicroRNAs
mRNAs: Messenger RNAs
circRNAs: Circular RNAs
lncRNAs: Long non-coding RNAs
EDTA-2K: Dipotassium ethylene diamine tetraacetic acid
T_1/2_: Half-life
qPCR: Real-time quantitative PCR
GAPDH: Glyceraldehyde-3-phosphate dehydrogenase
ANOVA: Multivariate analysis of variance
