# Supplementary material for: Factors influencing degradation kinetics of mRNAs and half-lives of microRNAs, circRNAs, lncRNAs in blood in vitro using quantitative PCR
Source: Sci Rep. 2022 May 4;12:7259. doi: 10.1038/s41598-022-11339-w (PMC9068688; doi:10.1038/s41598-022-11339-w)
Supplement: Supplementary file 1 — Supplementary Information. [file 41598_2022_11339_MOESM1_ESM.docx]

**Factors influencing** **degradation kinetics of mRNAs and half-lives of microRNAs, circRNAs, lncRNAs in blood in vitro using quantitative PCR**

**Running Head:** Degradation kinetics of mRNAs, microRNAs, circRNAs and lncRNAs

**Chong Wang^1^, Hui Liu***

College of Medical Laboratory, Dalian Medical University, Dalian 116044, China

Corresponding author:

Professor Hui Liu

*College of Medical Laboratory, Dalian Medical University, Dalian 116044, China

E-mail address: liuhui60@sina.com,

Tel.: +86 411 86110383, Fax: +86 411 86110392

**Supplementary materials**


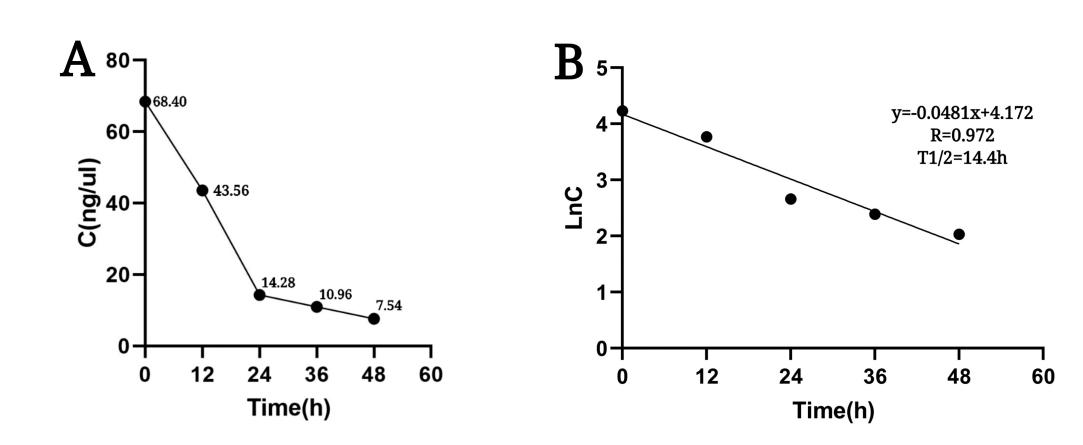


Figure S1 Dynamic degradation curves for RNA in blood before and after natural logarithm transformation.

**Original data：**

**Table 1** Linear detection using a high-efficiency blood RNA extraction kit (A).

| Dilution | Concentration (ng/μl) | Concentration (ng/μl) | Concentration (ng/μl) |
| --- | --- | --- | --- |
| 1.00 | 146.53 | 144.16 | 148.60 |
| 2.00 | 83.73 | 82.60 | 87.00 |
| 4.00 | 31.31 | 30.72 | 31.68 |
| 8.00 | 17.21 | 16.20 | 17.92 |
| 16.00 | 7.35 | 5.05 | 5.64 |

**Table 2** Linear detection by microspectrophotometry (B).

| Dilution | Concentration (ng/μl) | Concentration (ng/μl) | Concentration (ng/μl) |
| --- | --- | --- | --- |
| 1.00 | 112.50 | 42.16 | 132.12 |
| 2.00 | 55.80 | 21.26 | 65.20 |
| 4.00 | 27.32 | 11.00 | 32.40 |
| 8.00 | 12.20 | 5.64 | 15.08 |
| 16.00 | 5.44 | 2.92 | 7.96 |
| 32.00 | 2.16 | 1.64 | 4.28 |

**Table 3** Linear detection by real-time quantitative PCR amplification (C).

| Dilution | GAPDH (Ct) | GAPDH (Ct) | GAPDH (Ct) |
| --- | --- | --- | --- |
| 1.00 | 20.57 | 21.42 | 20.24 |
| 2.00 | 21.37 | 22.31 | 21.17 |
| 4.00 | 21.68 | 22.58 | 21.65 |
| 8.00 | 23.47 | 24.27 | 23.12 |
| 16.00 | 24.40 | 25.32 | 24.38 |
| 32.00 | 25.91 | 26.93 | 25.94 |

**Table 4 L_9_(3^4^) orthogonal experiment**

| Group | A | B | C | β-actin (Ct) |
| --- | --- | --- | --- | --- |
| 1.00 | 1.00 | 1.00 | 1.00 | 21.96 |
| 1.00 | 1.00 | 2.00 | 2.00 | 22.51 |
| 1.00 | 1.00 | 3.00 | 3.00 | 22.68 |
| 1.00 | 2.00 | 1.00 | 2.00 | 23.71 |
| 1.00 | 2.00 | 2.00 | 3.00 | 23.73 |
| 1.00 | 2.00 | 3.00 | 1.00 | 23.49 |
| 1.00 | 3.00 | 1.00 | 3.00 | 24.34 |
| 1.00 | 3.00 | 2.00 | 1.00 | 24.15 |
| 1.00 | 3.00 | 3.00 | 2.00 | 24.39 |
| 2.00 | 1.00 | 1.00 | 1.00 | 21.99 |
| 2.00 | 1.00 | 2.00 | 2.00 | 22.62 |
| 2.00 | 1.00 | 3.00 | 3.00 | 22.73 |
| 2.00 | 2.00 | 1.00 | 2.00 | 23.81 |
| 2.00 | 2.00 | 2.00 | 3.00 | 23.55 |
| 2.00 | 2.00 | 3.00 | 1.00 | 23.41 |
| 2.00 | 3.00 | 1.00 | 3.00 | 24.41 |
| 2.00 | 3.00 | 2.00 | 1.00 | 24.05 |
| 2.00 | 3.00 | 3.00 | 2.00 | 24.30 |
| 3.00 | 1.00 | 1.00 | 1.00 | 22.40 |
| 3.00 | 1.00 | 2.00 | 2.00 | 22.72 |
| 3.00 | 1.00 | 3.00 | 3.00 | 22.90 |
| 3.00 | 2.00 | 1.00 | 2.00 | 23.85 |
| 3.00 | 2.00 | 2.00 | 3.00 | 23.79 |
| 3.00 | 2.00 | 3.00 | 1.00 | 23.18 |
| 3.00 | 3.00 | 1.00 | 3.00 | 23.83 |
| 3.00 | 3.00 | 2.00 | 1.00 | 24.43 |
| 3.00 | 3.00 | 3.00 | 2.00 | 24.56 |

**Table 5 Changes in circRNAs concentration in whole blood at room temperature**

| Time (h) | hsa_circ_0000190 (Ct) | hsa_circ_0001785 (Ct) | hsa_circ_0000520 (Ct) | circARIDIB (Ct) | Circ002532 (Ct) |
| --- | --- | --- | --- | --- | --- |
| 0 | 28.19  28.32  28.30 | 29.11  29.08  29.13 | 27.35  27.38  27.32 | 28.22  28.17  28.31 | 26.43  26.64  26.40 |
| 12 | 29.19  29.22  29.17 | 29.29  29.32  29.34 | 27.82  27.84  27.79 | 28.54  28.41  28.56 | 26.62  26.59  26.73 |
| 24 | 30.28  30.17  30.31 | 29.91  29.88  29.94 | 28.02  28.21  28.13 | 28.91  29.04  29.10 | 27.98  27.72  28.04 |
| 36 | 30.58  30.56  30.61 | 30.83  30.84  30.78 | 28.65  28.54  28.66 | 30.02  30.12  29.98 | 28.63  28.57  28.64 |
| 48 | 31.24  31.22  31.34 | 30.89  30.94  31.03 | 28.82  28.97  29.01 | 30.12  30.08  30.21 | 28.72  28.68  28.81 |
| 60 | 31.88  31.86  31.94 | 31.13  31.09  31.18 | 29.22  29.19  29.23 | 30.47  30.45  30.52 | 28.73  28.70  28.88 |

**Table 6 Changes in lncRNAs concentration in whole blood at room temperature**

| Time (h) | lncRNA_PCGEM1 (Ct) | NR_038263 (Ct) | LncRNASNHG5(Ct) | STEAP3_AS1 (Ct) | LncRNA GASL (Ct) |
| --- | --- | --- | --- | --- | --- |
| 0 | 30.49  30.53  30.55 | 27.51  27.47  27.45 | 26.38  26.34  26.41 | 25.44  25.40  25.33 | 32.53  32.45  32.58 |
| 12 | 31.18  31.34  31.30 | 28.23  28.19  28.25 | 27.21  27.19  27.24 | 25.72  25.71  25.82 | 33.24  33.56  33.41 |
| 24 | 32.36  32.29  32.40 | 29.34  29.33  29.37 | 27.69  27.66  27.74 | 26.29  26.42  26.32 | 33.47  33.41  33.48 |
| 36 | 32.64  32.59  32.65 | 29.88  29.93  29.98 | 28.82  28.79  28.91 | 26.90  26.87  26.96 | 34.21  34.12  34.29 |
| 48 | 33.43  33.41  33.47 | 30.24  30.32  30.19 | 29.19  29.34  29.39 | 27.78  27.94  27.83 | 34.53  34.64  34.72 |
| 60 | 34.28  34.29  34.23 | 31.46  31.65  31.43 | 30.92  30.82  31.01 | 28.21  28.16  28.29 | 35.63  35.71  35.57 |

**Table 7 Changes in miRNAs concentration in whole blood at room temperature**

| Time (h) | miR-16-1  (Ct) | miR-28-3p  (Ct) | miR-126  (Ct) | miR-145  (Ct) | miR-221 (Ct) |
| --- | --- | --- | --- | --- | --- |
| 0 | 18.41  18.31  18.33 | 24.65  24.71  24.69 | 23.32  23.27  23.35 | 20.94  20.91  20.98 | 18.42  18.45  18.51 |
| 12 | 19.45  19.42  19.49 | 24.75  24.77  24.69 | 23.47  23.52  23.54 | 21.27  21.22  21.32 | 19.19  19.28  19.31 |
| 24 | 21.79  21.86  21.83 | 25.09  25.11  24.98 | 24.47  24.39  24.51 | 21.66  21.72  21.74 | 19.52  19.47  19.54 |
| 36 | 22.62  22.58  22.70 | 25.20  25.17  25.29 | 25.56  25.55  25.57 | 22.72  22.73  22.68 | 19.77  19.67  19.68 |
| 48 | 23.61  23.59  23.63 | 26.49  26.58  26.56 | 26.53  26.51  26.10 | 23.25  23.19  23.28 | 20.89  20.87  20.92 |
| 60 | 24.01  24.17  23.97 | 27.81  27.76  27.83 | 27.01  27.12  27.07 | 24.41  24.45  24.37 | 21.52  21.57  21.49 |
